# Unusual Complication of Intercostal Tube Drainage of Penetrating Chest Injury: A Case Report

**DOI:** 10.7759/cureus.13813

**Published:** 2021-03-10

**Authors:** Saumya Agrawal, Bhargav Gajula, Anil Kumar Chongtham, Farhanul Huda, Somprakas Basu

**Affiliations:** 1 Surgery, University of Illinois at Chicago, Chicago, USA; 2 Surgery, All India Institute of Medical Sciences, Rishikesh, Rishikesh, IND; 3 General Surgery, All India Institute of Medical Sciences, Rishikesh, Rishikesh, IND

**Keywords:** traumatic diaphragmatic injuries, traumatic diaphragmatic rupture, traumatic diaphragmatic hernia, penetrating diaphragmatic injuries

## Abstract

Penetrating chest injuries can lead to diaphragmatic injuries, which are often missed easily on initial assessments, especially in patients with polytrauma. We are usually more focused and biased towards other evident, immediately life-threatening injuries. The fact that clinical and radiological findings are subtle, especially on chest X-rays, which is sometimes the only investigation performed, highlights the importance of using higher imaging modalities in stable patients and that a clinician should be suspicious of this entity with the corresponding history. Intervening in such patients with the placement of intercostal drain can contribute to morbidity and mortality, as in our case, by causing inadvertent injury to the herniating structures. The case report briefs the same and emphasizes that thoracic injuries, especially penetrating ones, should ring a bell and should be carefully investigated further before the intervention.

## Introduction

Penetrating injuries to the chest are usually associated with hemothorax or pneumohemothorax, and the standard of care is the placement of an intercostal chest drain [1]. However, although drains are mostly associated with complications like bleeding from intercostal vessels, injury to the lung, or long thoracic nerve, if an underlying diaphragmatic injury is present and has been missed, the drain may injure the herniating hollow viscus, leading to life-threatening complications. This injury can easily be missed, both clinically due to lack of specific physical findings and on basic imaging, including chest X-rays, especially in the presence of usually related hemothorax or pleural effusions, atelectasis, due to poor quality of imaging, or inexperienced healthcare providers [2]. Hence, a high index of suspicion should be kept before any intervention such as the placement of an intercostal drain, which may prove life-threatening, as in the presenting case.

There have been several case reports on blunt and penetrating injuries to the diaphragm followed by a hernia and associated complications [3]. However, an extensive review of literature has not revealed any case of multiple perforations of the diaphragm following a single penetrating injury, the condition being worsened by the inadvertent injury to the herniating abdominal viscera by the placement of an intercostal drain; this paucity of data on such cases has led us to present this case report.

## Case presentation

A 30-year-old laborer presented to the emergency department with the complaint of the passage of food in the chest drain placed in the left side of his chest, associated with upper abdominal pain, chest pain, and respiratory distress for eight days. He had a history of fall from a height of about 20 feet followed by penetration of a steel plate into his left chest eight days back, which his co-workers had removed on the spot. He had been treated at a local hospital with the placement of a left intercostal chest drain in the left fifth intercostal space in the midaxillary line. The drain had been manipulated when the patient had developed the current symptoms on the next day of insertion and had been changed at another hospital but to no relief.

**Figure 1:**
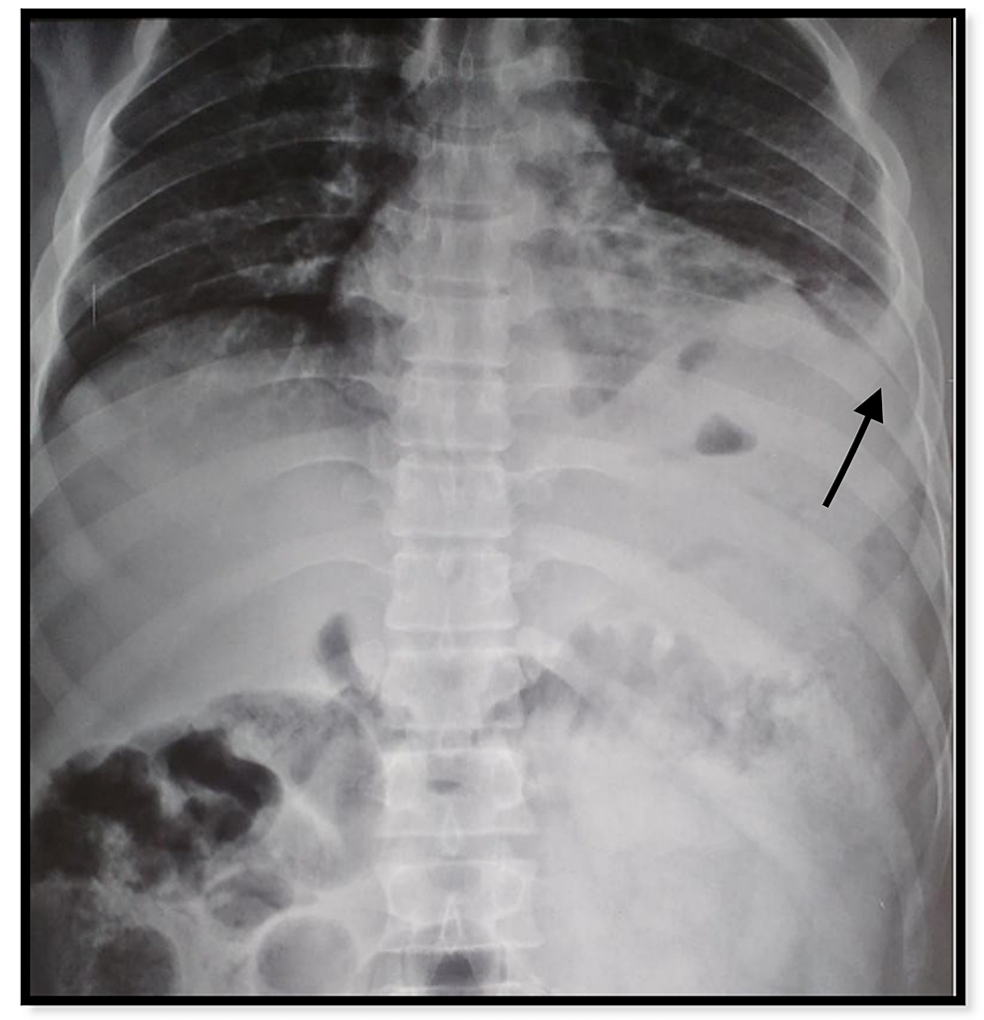
The initial X-ray at the time of injury The injury was misinterpreted as left hemothorax (black arrow)

At the time of presentation, the patient was tachypneic, although hemodynamically stable. He was maintaining his oxygen saturation at 95% on room air but with bilateral wheeze and crepitations on the left, tenderness in the upper abdomen more in the left hypochondrium, and food particles in his chest drain, which was in situ in the left fifth intercostal space. There was no associated injury. Complete blood counts revealed leukocytosis, 33,900/Cu mm, with 94% neutrophilia. Liver function tests showed raised total bilirubin of 2.15 mg/dl and decreased total protein and total albumin. Renal function tests revealed raised urea levels of 53 mg/dl. A chest X-ray revealed blunting of left costophrenic angle. The patient's initial chest X-ray is presented below (Figure 1).

**Figure 2:**
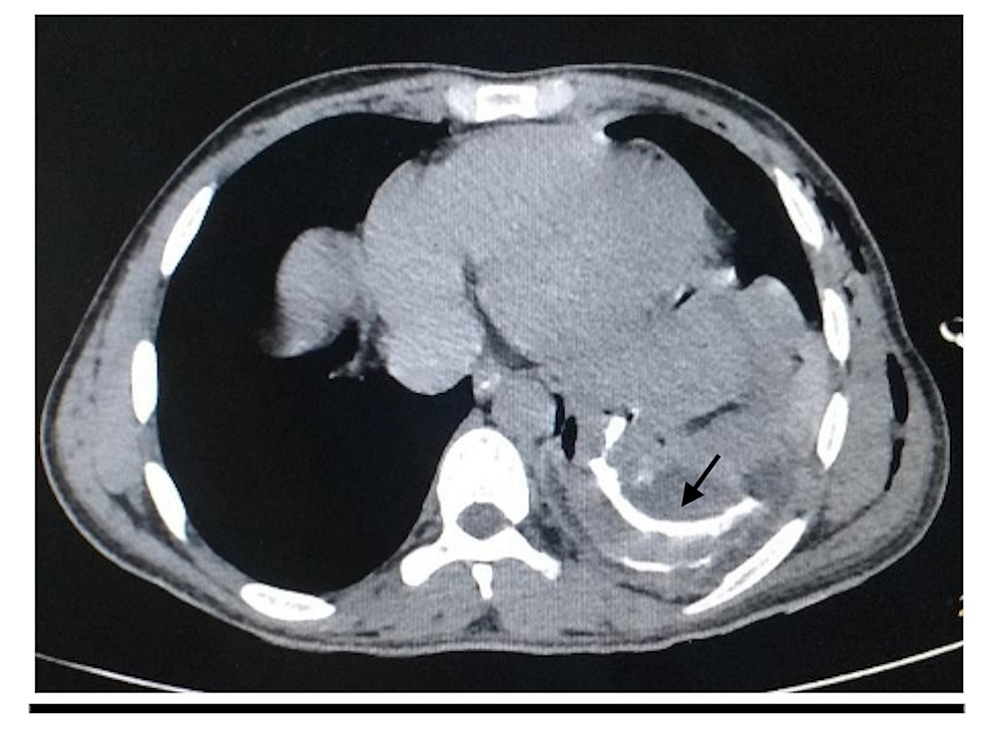
The CT scan done at the time of presentation The image shows herniated abdominal viscera with oral contrast in the left hemithorax (black arrow) CT: computed tomography

Ultrasonography of the abdomen was suggestive of mild ascites and loculated left pleural collection. Contrast-enhanced CT of the thorax was indicative of a focal defect of 29 mm in the dome of the left hemidiaphragm. The fundus of the stomach was seen herniating into the left pleural cavity, with moderate left hydro-pneumothorax and the passive collapse of the left lower lobe (Figure 2).

On oral contrast administration, leakage of contrast occurred through a defect in the posterior wall of the stomach into the pleural cavity.

**Figure 3:**
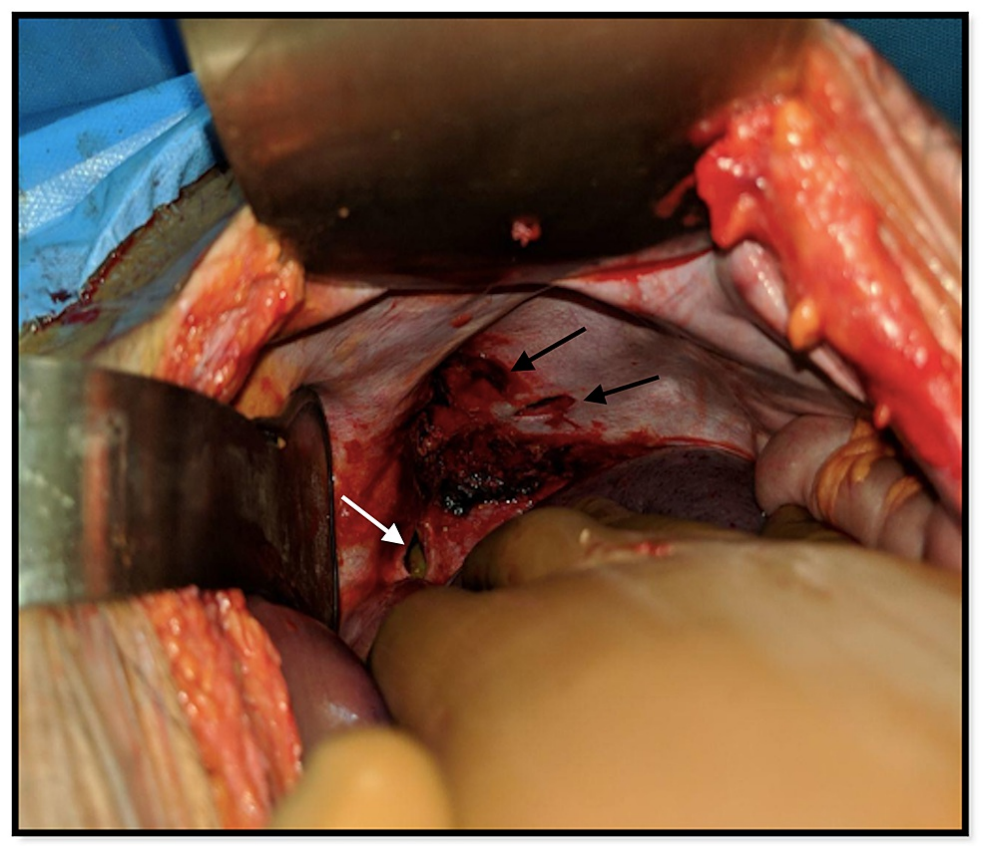
Intraoperative image showing two openings in the left dome of diaphragm (after the reduction of herniating contents): inferomedially (white arrow) and superolaterally (black arrows)

The patient underwent exploratory laparotomy. The intraoperative findings showed two diaphragmatic openings in the left dome. One of them was 4 x 1 cm in size and present inferomedially with the fundus of the stomach herniating through it, and the other was 3 x 1 cm in size and present inferolaterally with omentum herniating through it (Figure 3).

**Figure 4:**
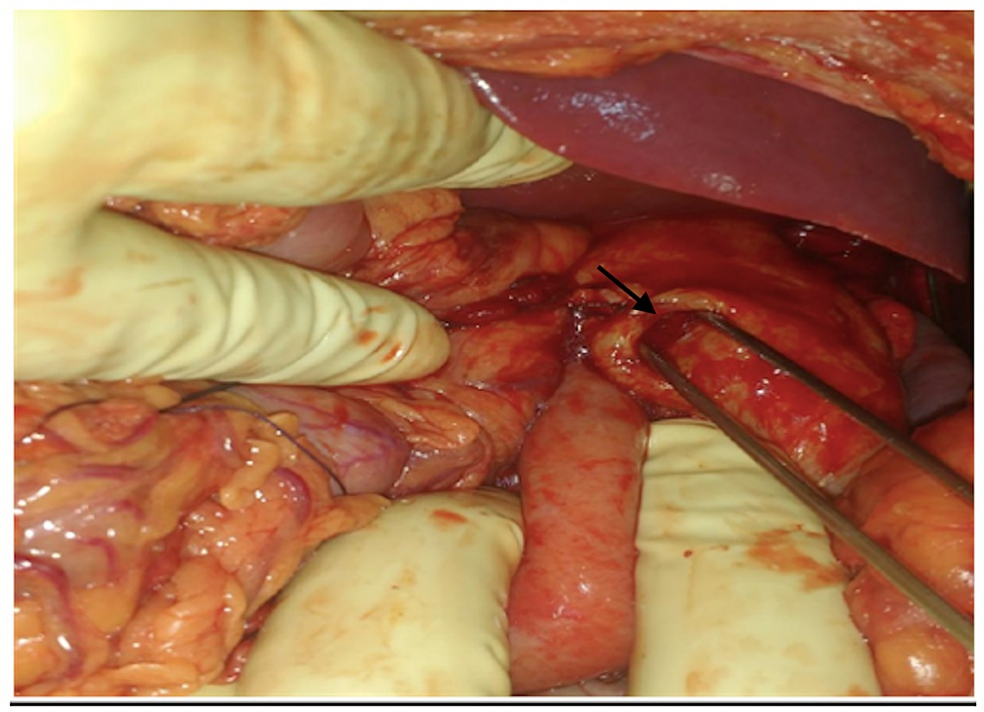
The perforation in the fundus of the stomach (black arrow) that was herniating through the diaphragmatic opening into the thorax

The omentum was found to be sutured to the traumatic wound on the chest that was released. The herniating contents were reduced, and a lacerated wound of 1 x 1 cm in size on the fundus of the stomach was noted (Figure 4).

The diaphragmatic openings were closed with no. 1 Prolene with mattress sutures. The fundal opening was repaired by the Cellan-Jones technique where, after freshening the margins, a pedicled omentum was placed. Feeding jejunostomy was also done.

Postoperatively, the patient showed symptomatic improvement over the next few days; feeding via jejunostomy was started on day three. He had purulent drainage in his chest drain for the first few days. The use of empirical antibiotics piperacillin-tazobactam, metronidazole, and amikacin helped decrease the output to 250 ml on day eight from 500 ml on day one, besides changing the nature to serosanguinous type.

Culture grew *Acinetobacter* and methicillin-resistant *Staphylococcus aureus* (MRSA) sensitive to amikacin and linezolid, respectively, and antibiotics were changed accordingly. Serial chest X-rays were done to assess the improvement. Incentive spirometry, aggressive chest physiotherapy, and maintenance of nutrition eventually led to the rapid recovery of the patient.

The patient was followed up weekly after discharge for a month, and the drain was removed when the output was minimal, and the chest skiagram showed satisfactory expansion of the lung. He has been followed up once every three months since then for a year and has been doing fine.

## Discussion

Chest injuries, including penetrating ones, are usually associated with complications such as pneumothorax, hemothorax, hemopneumothorax, tamponade, emphysema, cardiac rupture, pericardial effusion, and pleural effusion, in the order of frequency. It is reported that supportive treatment and chest tube placement are adequate for 90-95% of these patients, and thoracotomy is required in less than 10% of patients. However, diaphragmatic injuries are also one of the complications, and they are easily missed and overlooked in the initial assessments, especially in patients with multiple injuries.

As per our present knowledge of diaphragmatic injuries, they account for only 1% of all traumatic injuries [2], out of which 20% are due to penetrating injuries. The diaphragmatic ruptures are subtle and widely varied in their presentation and can give a roller coaster ride to both the patient and the clinician over days to months to years. Our patient presented eight days post-injury and required prolonged care to recover completely.

These injuries are so notorious that they have been known to be missed even on exploratory laparotomy following trauma and, surprisingly, even after explicitly being looked for [3]. Chest X-ray is the first and sometimes the only investigation performed, as in our patient, especially in a resource-poor setting, for any trauma to the thorax. However, several case series have reported the subtle inability of these X-rays to detect the injury in most cases [4]. In our case, the injury mimicked hemothorax and hence guided the further plan of action. We recommend that any suspicious finding be confirmed by a higher imaging modality like CT scan, though they have been estimated to miss 30-50% of diaphragmatic injuries during the initial assessment as well [5]. Thoracoscopy and laparoscopy have been advocated as they have both diagnostic and therapeutic relevance. The management is undoubtedly surgical, but the approach varies according to the surgeon's choice and expertise and also depending on the type and number of injuries and the clinical condition of the patient. The traditional laparotomy is still the most preferred method, while thoracotomy is chosen in cases of late diaphragmatic hernia, isolated lesions of the right diaphragm, and in cases of suspicion of chest injury [6]. The reason why the proper identification of the injury and careful intervention are being stressed here is that they help avoid the natural course of complications that follow [7]. A high index of suspicion is necessary to avoid the iatrogenic injury such as the one that occurred in our case owing to the placement of an intercostal drain, while the stomach was herniating in the thoracic cavity, which had been done in an attempt to drain the suspected hemothorax on the chest skiagram.

## Conclusions

As demonstrated in this case report, the crucial role of more focused and further investigations before any intervention, particularly in a hemodynamically stable patient with suspected hemothorax, cannot be overstated. The message is loud and clear: thoracic injuries, especially penetrating ones, should ring a bell and raise suspicion of diaphragmatic injury.
